# A promising form-stable phase change material prepared using cost effective pinecone biochar as the matrix of palmitic acid for thermal energy storage

**DOI:** 10.1038/s41598-019-47877-z

**Published:** 2019-08-08

**Authors:** Ye-chao Wan, Yan Chen, Zhi-xing Cui, Han Ding, Shu-feng Gao, Zhi Han, Jun-kai Gao

**Affiliations:** 1 School of Port and Transportation Engineering, Zhejiang Ocean University, Zhoushan, 316022 China; 2 Yinzhou Kefeng New Material of Polymer Co. Ltd., Ningbo, 315100 China

**Keywords:** Energy, Sustainability

## Abstract

A promising new form-stable phase change material (PA/PB) was fabricated using pinecone biochar (PB) as the supporting material of palmitic acid (PA). The biochar of PB with large surface area was produced by forest residue of pinecone, and it was cheap, environment friendly and easy to prepare. The PB was firstly utilized as the supporter of PA and the characterizations of PA/PB were analyzed by the BET, SEM, XRD, DSC, TGA, FT-IR and thermal conductivity tester. The results demonstrated that the PA was physically absorbed by the PB and the crystal structure of the PA was not destroyed. The results of DSC showed that the fusing and crystallization points of the form-stable phase change material with the maximum content of PA (PA/PB-4) were 59.25 °C and 59.13 °C, and its fusing and freezing latent heat were 84.74 kJ/kg and 83.81 kJ/kg, respectively. The results of TGA suggested that the thermal stability of the PA/PB-4 composite was excellent, which could be used for the applications of thermal energy storage. Furthermore, the thermal conductivity of PA/PB-4 was 0.3926 W/(m∙K), which was increased by 43.76% compared with that of the pure PA. Thus, the study results indicated that the PA/PB-4 had great potential for thermal energy storage applications.

## Introduction

In recent years, phase change materials (PCMs) have been studied and applied extensively all over the world because of their advanced properties, such as small size change, high latent heat density and sustained phase change temperature^1–4^. Moreover, using PCMs can enhance energy utilization of the system^5^. PCMs are classified into three categories: inorganic PCMs, organic PCMs and eutectic PCMs^6^. Compared to the other two PCMs, organic PCMs are non-corrosive, and have high thermal stability and large latent heat^5,7^.

Palmitic acid (PA), as one of the ideal PCMs, has many merits such as little or no supercooling phenomenon, chemical stability, large latent heat and appropriate fusing point^8,9^. Therefore, a lot of researchers are attracted to investigate its application in many fields, such as smart housing, textiles and temperature-control greenhouse^10,11^. However, there are two disadvantages about PA that should be overcome. One is that the thermal conversion would be weakened as a result of the low thermal conductivity of the PA. The other is that the leakage usually occurs during the phase change processes^12^. These drawbacks will limit the practical application of PA^13^. In order to resolve the above problems, the shape-stabilized PCMs composites were prepared^14,15^. In the shape-stabilized PCMs composites, the organic PCMs could be encapsulated by some inorganic materials, which have porous structure, large surface area and great thermal stability, such as mesoporous silica, activated carbon, diatomite and so on^16–18^. Han *et al*. used the mixture of nano-SiO_2_ and expanded graphite (EG) to encapsulate the paraffin under the high-temperature refining condition. The results showed that the paraffin was absorbed in nano-SiO_2_ and porous EG very well, and no leakage of melting paraffin was observed from the mixture even at 200 °C for 1 hour^19^. Sharma *et al*. used TiO_2_ nanoparticles as the matrix to fabricate palmitic acid/TiO_2_ composite, and the results suggested that the thermal conductivity of palmitic acid/TiO_2_ was increased by 80% than that of the pure palmitic acid when the nanoparticle weight fraction was 5%^5^. Wen *et al*. used diatomite as the supporting material to absorb the eutectic PCMs of capric acid and lauric acid. The shape-stabilized PCMs was heated to 80 °C and refrigerated to 15 °C as a whole thermal cycle, and they found that the melting and freezing enthalpy changed slightly after multiple thermal cycles, which indicated that the shape-stabilized PCMs had high thermal reliability^20^.

Activated carbon, which is derived from charcoal, has the merits of low density, high specific surface area, wide availability and great chemical stability^21^. Furthermore, it could hold the PCMs at stable-state and improve the thermal conversion performance^22^. Therefore, researchers paid much attention to study using activated carbon as the supporting material of PCMs. Chen *et al*. used activated carbon (AC) as the supporter of lauric acid (LA) to synthesize shape-stabilized material of LA/AC, and they found that lauric acid could be adsorbed into the activated carbon very well and the thermal conductivity of LA/AC was improved obviously compared to the pure LA^23^. Feng *et al*. used mesoporous activated carbon to prepare shape-stabilized phase change material of PEG/AC, and the results indicated that the activated carbon played a major role in preventing the leakage of PEG during the phase change process^24^. Yuan *et al*. prepared the shape-stabilized PCMs using capric-palmitic-stearic acid (CA-PA-SA) and activated carbon. They found that the heat storage time and heat release time of the shape-stabilized PCMs were reduced by 37% and 67% than that of the CA-PA-SA, which was attributed to the improvement of the thermal conductivity of shape-stabilized PCMs by the addition of activated carbon^25^.

However, the activated carbon has some shortcomings, such as high cost, difficult to regenerate and unrenewable^26^. Biochar, which is produced from biomass residues such as forest or agriculture residues^27^, is cheaper, easy synthesization and renewable^28^. Additionally, the utilization of biochar can protect environment, because it even could be produced from food wastes^29^. Furthermore, the biochar also has high surface area and large sorption capacities. Therefore, a mass of studies about biochar fabrication and utilization were carried out in recent years. Wang *et al*. used loblolly pine wood and citrus wood to prepare biochar, and they found that the surface area of loblolly pine wood biochar (LPB) and citrus wood biochar (CTB) could be as high as 209 m^2^/g and 182 m^2^/g, respectively^30^. Zhang *et al*. synthesized biochar by pyrolyzing celery-derived to absorb the Pb^2+^, and the results indicated that the adsorption capacity of Pb^2+^ by the biochar could be as high as 304 mg/g^31^. Sewu *et al*. pyrolyzed rice straw to fabricate the biochar of RS, and they found that the adsorption capacity of RS for cationic dye and crystal violet was higher than that of the activated carbon^32^. However, up to now, the study reports about utilizing biochar as the supporter of shape-stabilized phase change materials are still rare, and therefore, ongoing efforts are needed to study on the immobilization of organic phase change materials in the biochar.

Pinecone, as a residuum of pine tree, is very abundant and fairly easy to obtain in China, and therefore the price of pinecone is very cheap. Moreover, it is occasionally burned by the local people because of its limited practical application value, which could cause the air pollution^33^. Hence, the study on fabrication and utilization of biochar from pinecone is very meaningful. In this paper, the biochar (PB) was prepared from pinecone, and then it was firstly used as the supporting material of palmitic (PA) to synthetize shape-stabilized phase change material (PA/PB). The morphology and structural properties of PA/PB and its thermal properties were studied. The results indicated that the PA/PB possessed high performance in latent heat storage and had great potential in practical applications.

## Materials and Methods

### Materials

Pinecone was obtained from Quanzhou, Fujian Province, China. PA was purchased from Sinopharm Chemical Reagent Co., Ltd, and its melting temperature was 62–64 °C. All other reagents used in the experiment were of AR grade.

### Synthesis of PB

**Figure 1:**
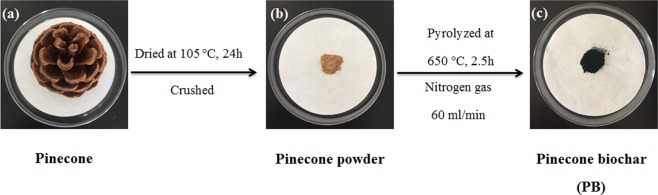
Schematic diagram of PB synthesization.

Figure 1 shows the schematic diagram of PB synthesization. The pinecone was washed by distilled water and then it was placed into the oven for 24 h at 105 °C. Then, the dried sample was crushed into powders. After that the pinecone powders were pyrolyzed at 650 °C for 2.5 h in a tube furnace with the protective gas (a nitrogen with the purity of 99.99%) at the flow rate of 60 ml/min. The as-prepared sample was PB.

### Synthesis of PA/PB

**Figure 2:**
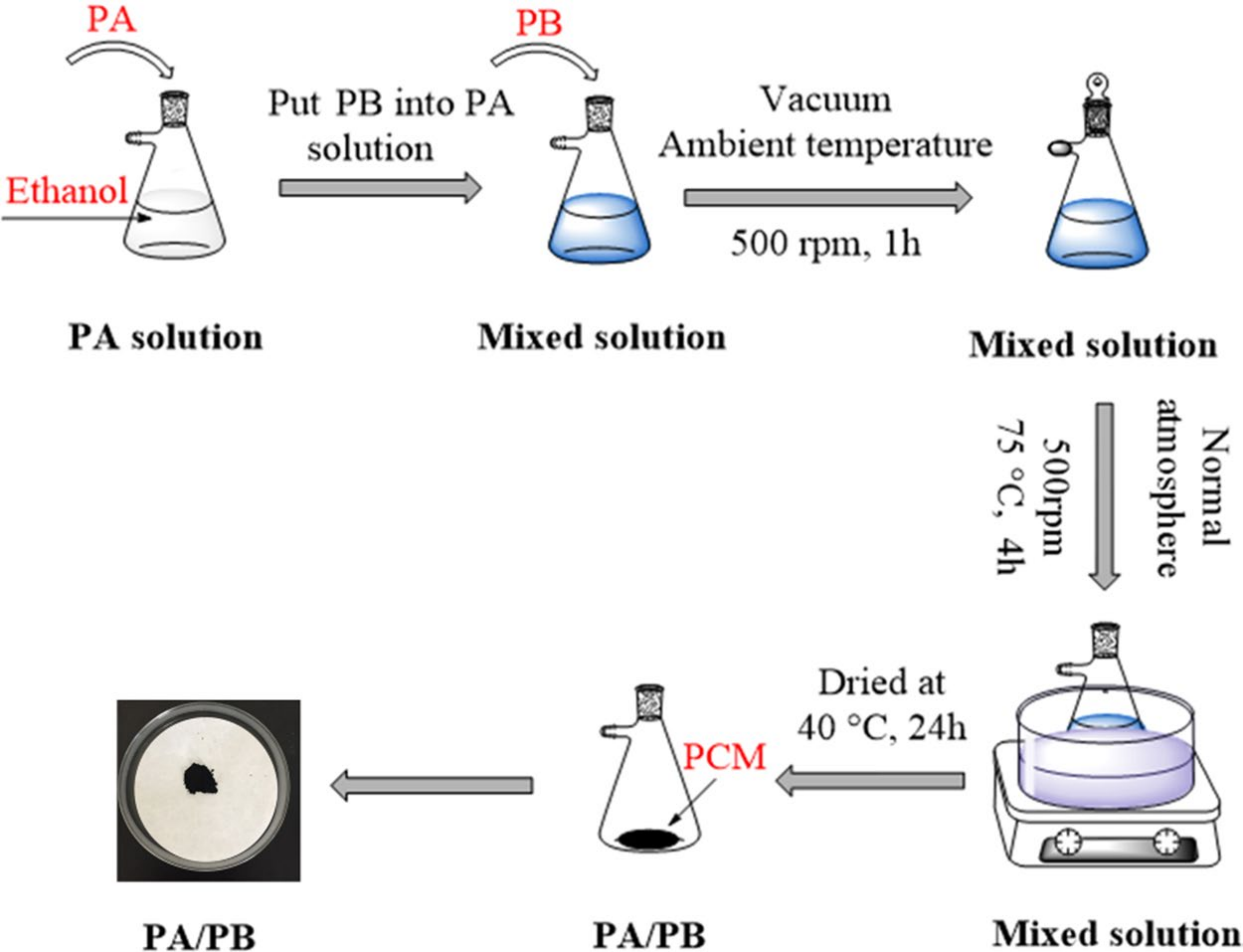
Schematic diagram of PA/PB synthesization.

Figure 2 shows the schematic diagram of PA/PB synthesization. The PA/PB was prepared using vacuum impregnation method. Firstly, PA was dissolved into 10 ml absolute ethanol in the conical bottle. Secondly, PB was put into the conical bottle. Then the blend was agitated with 500 rpm at ambient temperature under a vacuum atmosphere. After 1 h, the conical bottle was moved to the water-bath for 4 h at 75 °C under normal atmosphere. In the following, the composite was put in the oven at 40 °C overnight. Finally, the composite was regarded as a shape-stabilized composite if there was no leakage of melting PA from the mixture. In this study, the PA and PB were weighted with different mass ratios of 3:7, 4:6, 5:5, 6:4 and 6.5:3.5, and the corresponding composites were designated as PA/PB-1, PA/PB-2, PA/PB-3, PA/PB-4 and PA/PB-5.

### Characterization

The leakage test of the pure PA and PA/PB composites were carried out by putting them into an oven for 15 minutes at 75 °C and then they were observed whether there was any leakage of melted PA from the testing samples. The pore volume, pore diameter and surface area of the PB were measured by Brunauer-Emmett-Tellera (BET, Nova 2000e). The morphological images of PB and PA/PB composites were obtained by using the Scanning Electron Microscope (SEM, Quanta FEG 250). The crystalloid phase of the PA and the PA/PB composites were measured by the X-ray diffractometer (XRD, DX2700). The fourier transform infrared patterns of the PA and the PA/PB composites were obtained by the fourier transform infrared spectroscopy (FT-IR, Bruker, VECTOR22). The thermal stability and weight loss were measured on a thermogravimetric analysis (TGA, NETZSCH, STA449F3), and the testing temperature was from 50 °C to 500 °C with heating rate of 10 °C/min under protective gas of high-purity nitrogen (purity of 99.99%). The latent heat and phase change temperature of PA and PA/PB composites were investigated by using a differential scanning calorimetry (DSC, NETZSCH, 200-F3), and the heating and cooling rates were 10 °C/min with the temperature range between 0 to 100 °C under the protective gas of high-purity nitrogen (purity of 99.99%). The thermal conductivity of the PA and PA/PB composites were tested by the thermal conductivity meter (Foreda, CTPS-2500).

## Results and Discussion

### Leakage test of PA/PB

**Figure 3:**
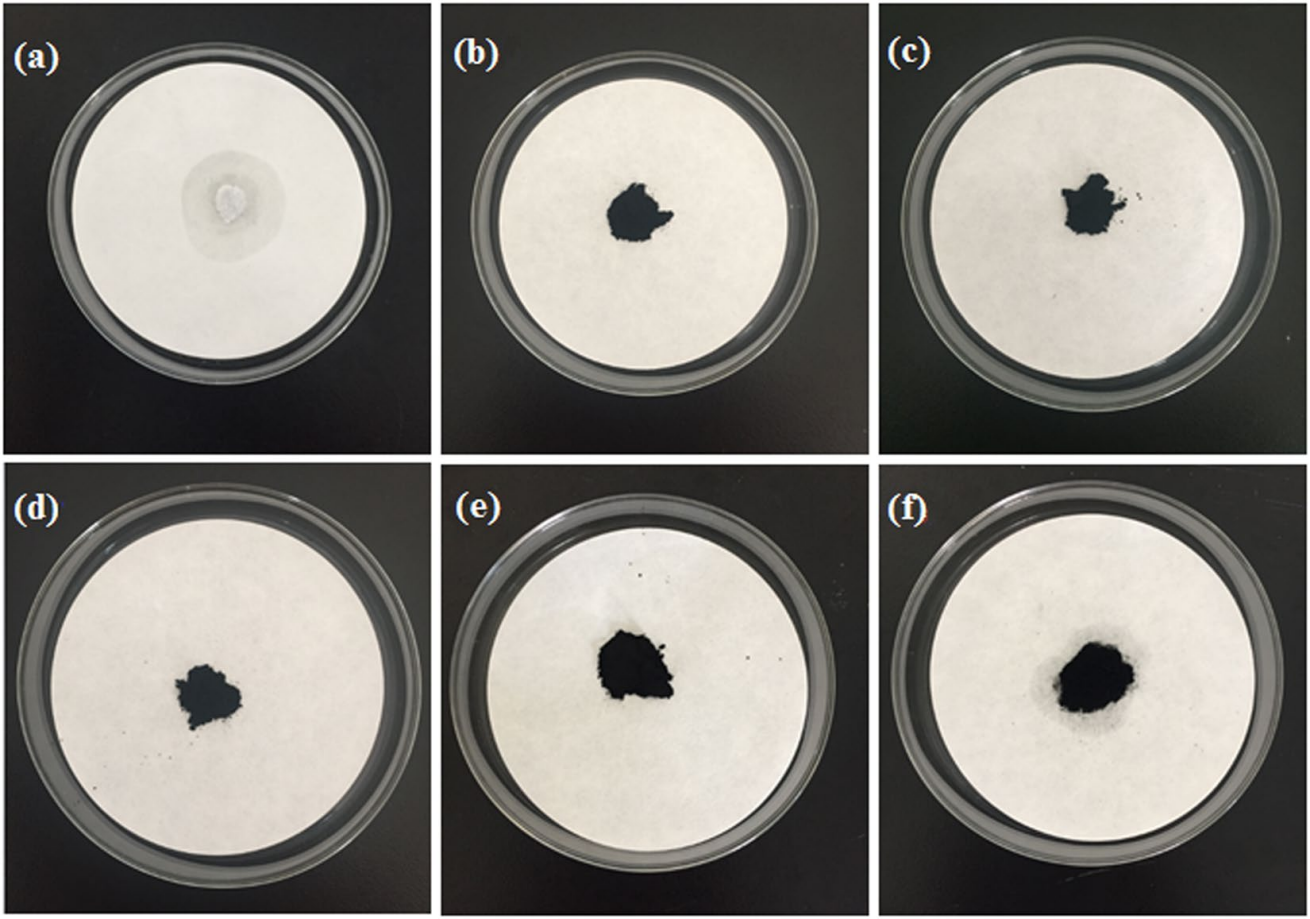
Leakage test of (**a**) PA, (**b**) PA/PB-1, (**c**) PA/PB-2, (**d**) PA/PB-3, (**e**) PA/PB-4 and (**f**) PA/PB-5.

The leakage test results of the pure PA and PA/PB composites are shown in Fig. 3, and it was clear that the pure PA melted at 75 °C (Fig. 3a) and no leakage were observed for PA/PB-1 (Fig. 3b), PA/PB-2 (Fig. 3c), PA/PB-3 (Fig. 3d) and PA/PB-4 (Fig. 3e) composites, while the PA/PB-5 composite (Fig. 3f) had a spot of leakage at 75 °C. Hence, the PA/PB-4 composite was used as the main research object in the following experiments for the study of thermal properties of form-stable phase change material.

### Characterization of PB and PA/PB-4

**Figure 4:**
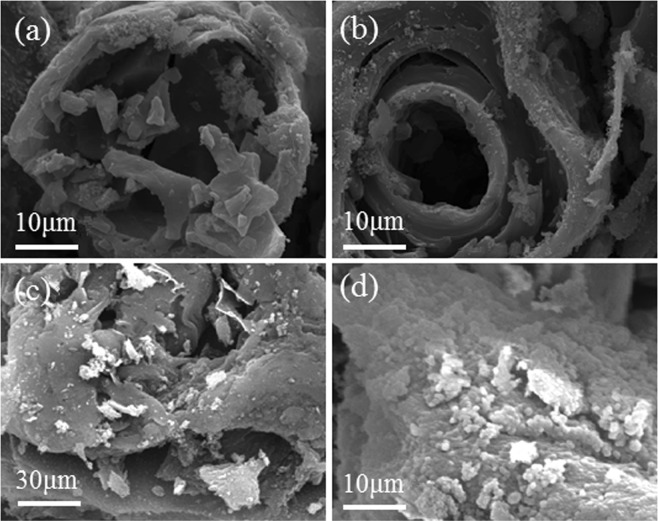
SEM images of (**a**,**b**) PB and (**c**,**d**) PA/PB-4.

**Figure 5:**
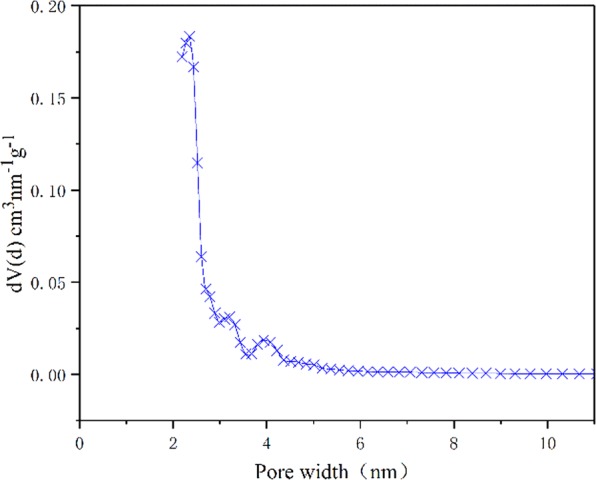
Pore size distribution curve of the PB.

Figure 4 presents the SEM photographs of the PB and PA/PB-4 composite. It was known from Fig. 4(a,b) that PB possessed porous structures, and its pores were open to surface which could adsorb the phase change material. Figure 4(c,d) illustrate the SEM photographs of PA/PB-4 composite. In the images, it could be seen that PA was well adsorbed and filled in the PB. In Fig. 5, it could be found that there were three palpable peaks at 2.3 nm, 3.2 nm and 4.0 nm and the average pore diameter was 3.078 nm, which suggested that the PB was mesoporous biochar. The pore volume and surface area of PB were 0.17cc/g and 289.69 m^2^/g. Additionally, the structural characteristic parameters of biochars prepared from different plants are shown in Table 1. The results indicated that the PB prepared in this study was competitive, and the large pore volume and high specific surface area of PB were beneficial for the PA/PB composite to preventing the leakage of melted PA in the phase change process.

**Table 1:** The structural parameter of different biochars.

Sample name	Pore volume (cc/g)	Pore diameter (nm)	Surface area (m^2^/g)	References
Rapeseed stem biochar	0.1052	2.20	316.90	Zhao, *et al*.^43^
Maize straw biochar	0.06		70.00	Wang, *et al*.^44^
Castor oil cake biochar	0.00314	6.9115	1.82	Kalinke, *et al*.^45^
Pinecone biochar	0.17	3.078	289.69	Present study

### XRD patterns of PB and PA/PB-4

**Figure 6:**
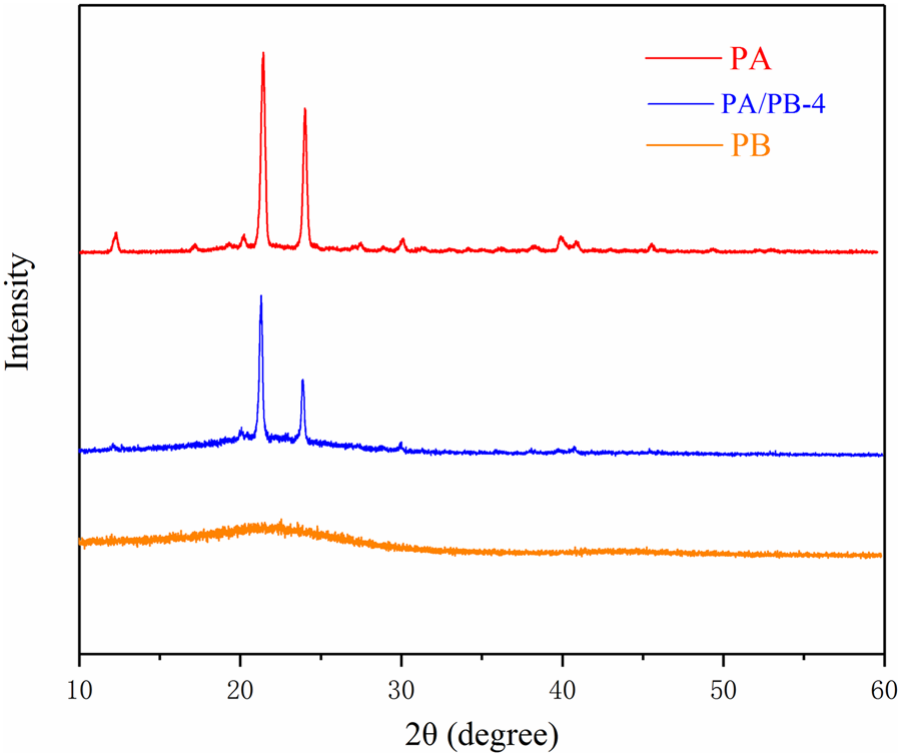
XRD patterns of the PA, PA/PB-4 and PB.

**Figure 7:**
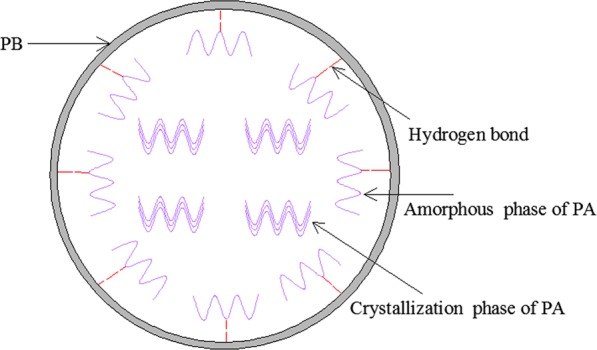
Schematic diagram of the interaction between PA and PB.

Figure 6 exhibits the X-ray diffraction curves of pure PA, PB and PA/PB-4 composite. The XRD pattern of PB shows that there was a wide arc-shaped peak within the scope of 18.3–27.7°, which indicated that PB had an amorphous structure^23^. The XRD pattern of PA indicates that there were two sharp peaks at 22.3° and 24.2°, which were attributed to the regular crystallization of PA^9^. It could also be seen from the XRD pattern of PA/PB-4 that the sharp diffraction peaks at 22.3° and 24.2°appeared in PA/PB-4 composite, which suggested that no chemical reactions happened between PA and PB, and the crystal structure of PA was not changed^5^. However, the X-ray peaks at 22.3° and 24.2° of the PA/PB-4 composite were lower than that of the pure PA. It could be explained that a few crystal growth of PA in the PA/PB-4 composite was limited by the pores of PB because of the hydrogen bond between the PA and PB^34^, and the schematic diagram of the interaction between the PA and PB is shown in Fig. 7.

### FT-IR analysis of PA, PB and PA/PB-4

**Figure 8:**
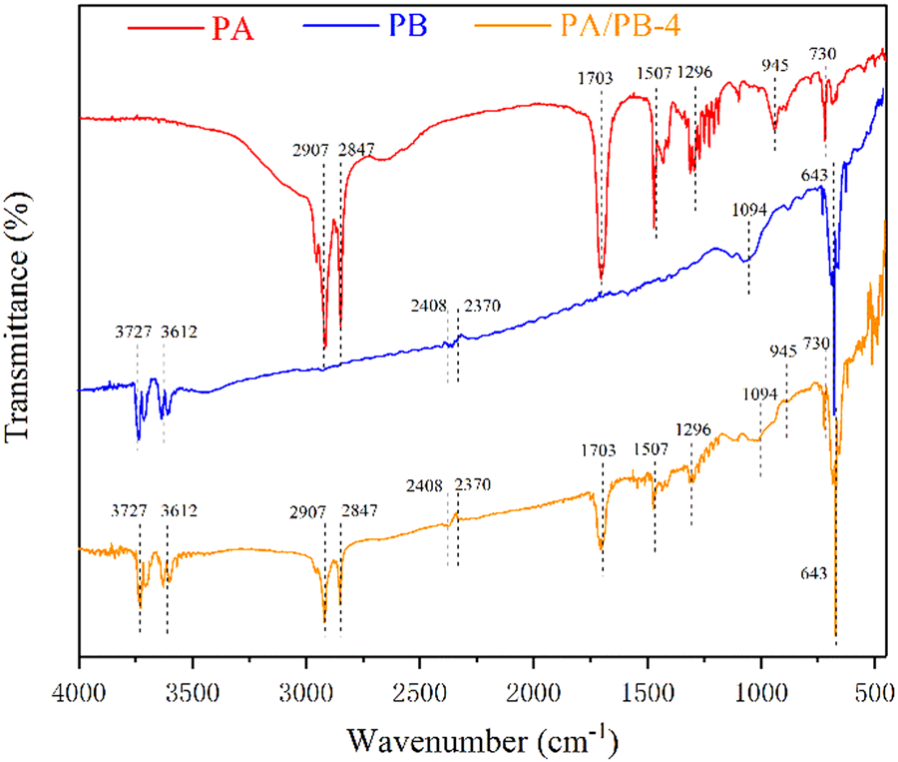
FT-IR spectra of PA, PB and PA/PB-4.

The FT-IR spectra of PA, PB and PA/PB-4 between the wave numbers of 450 cm^−1^ and 4000 cm^−1^ are shown in Fig. 8. As seen from the spectrum of PA, the strong peaks at 2907 cm^−1^ and 2847 cm^−1^ signified the symmetrical stretching vibration of –CH_3_ and –CH_2_ groups^5^. The strong peak at 1703 cm^−1^ was corresponded to the C=O stretching vibration^5^. The peak at 1507 cm^−1^ represented the –CH_3_ deformation vibration^5^. The absorption peaks of 1296 cm^−1^ and 945 cm^−1^ were corresponded to the in-plane bending vibration and out-plane bending vibration of –OH group^35^. The peak at 730 cm^−1^ was due to the in-plane swinging vibration^35^. As can be seen from the spectrum of PB, two strong peaks observed at 3612 cm^−1^ and 3727 cm^−1^ showed the N–H and O–H stretching vibrations in protein or cellulose compounds^36^. The peak at 2370 cm^−1^ was assigned to the C–H stretching vibration of the aldehyde groups^37^. The peak at 2408 cm^−1^ was attributed to the amine groups of proteins N-H stretch^36^. The absorption peak observed at 1094 cm^−1^ was assigned to the stretching vibration of C–O functional groups^37^. The peak at 643 cm^−1^ was due to the O–H out-plane bending vibration^38^. It could be clearly seen from the spectrum of PA/PB-4that the peaks at 2907 cm^−1^, 2847 cm^−1^, 1703 cm^−1^, 1507 cm^−1^, 1296 cm^−1^, 945 cm^−1^ and 730 cm^−1^ in the spectrum of PA all appeared in the spectrum of PA/PB-4 composite, and compared to the spectra of PA and PB, no new peak was found in the spectrum of PA/PB-4. The result suggested that there was no chemical reaction between the PA and PB, and the interaction between the PA and PB was mainly attributed to the surface tension and capillary force between the PA and porous PB^39^.

### Thermal stability of PA and PA/PB-4

**Figure 9:**
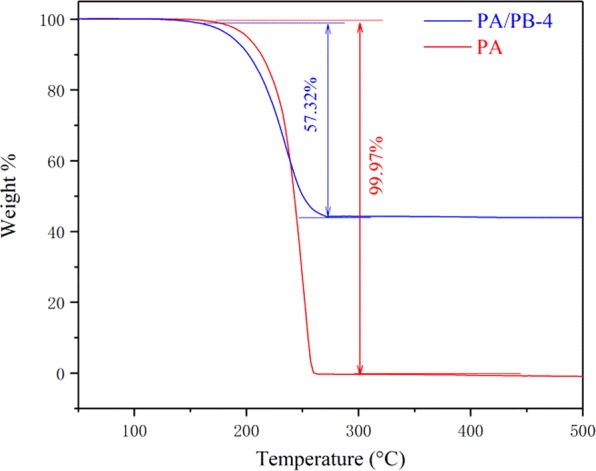
TGA curves of PA and PA/PB-4.

Thermal stability was a significant parameter for the form-stable phase change materials in latent heat storage applications, and it could be measured by the TGA. As can be seen from the TGA curves of PA and PA/PB-4 in Fig. 9, the processes of weight loss of the pure PA and PA/PB-4 composite occurred in only one step, and the PA started to decompose at 168 °C and stopped at 257 °C. As for the PA/PB-4 composite, the weights loss processes occurred between 152 °C and 273 °C with 57.32 wt% PA lost, and therefore, the PA/PB-4 composite had favorable thermal stability below 152 °C, which was beneficial to the practical application of PA/PB-4 in solar based thermal energy storage^40^.

### Thermal properties of PA/PB composites

**Figure 10:**
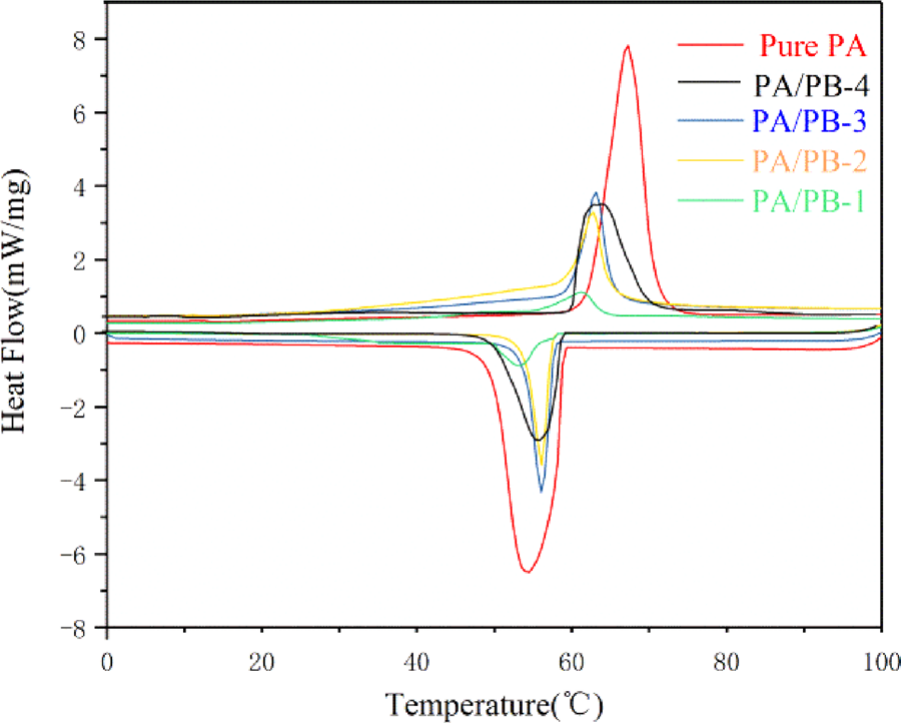
DSC curves of PA and PA/PB composites.

**Figure 11:**
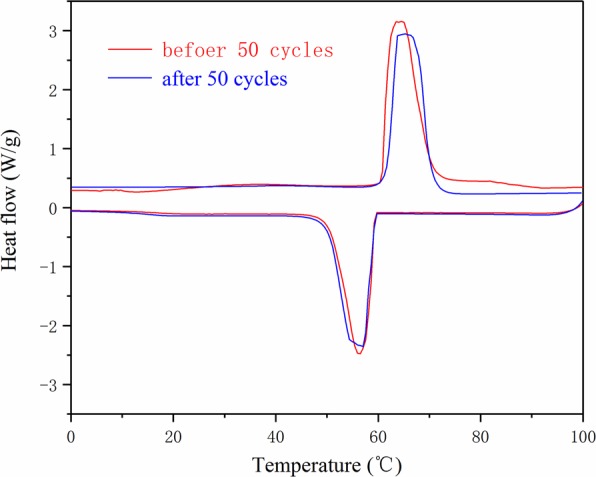
The enthalpy curves of the PA/PB-4 before and after 50 cycles.

The DSC results of the PA/PB-1, PA/PB-2, PA/PB-3, PA/PB-4 and pure PA are shown in Figs. 10, 11 and Table 2. It can be clearly seen in Fig. 10 and Table 2, the fusing temperatures of the PA and PA/PB-1, PA/PB-2, PA/PB-3, PA/PB-4 were determined to be 62.07 °C, 57.49 °C, 58.11 °C, 58.77 °C and 59.25 °C, respectively. The freezing temperatures of the PA and PA/PB-1, PA/PB-2, PA/PB-3, PA/PB-4 were determined to be 61.79 °C, 57.33 °C, 57.61 °C, 58.24 °C and 59.13 °C, respectively. It could be known that the fusing temperatures and freezing temperatures of the pure PA was higher than the PA/PB composites, which might be attributed to that in the PA/PB composite the crystalline region of PA became smaller while PB as the impurity^24^. As shown in Table 2, the fusing latent heats of the PA and PA/PB-1, PA/PB-2, PA/PB-3, PA/PB-4 were measured to be 219.63 kJ/kg, 14.88 kJ/kg, 43.62 kJ/kg, 58.56 kJ/kg and 84.74 kJ/kg, respectively. The solidifying latent heats of the PA and PA/PB-1, PA/PB-2, PA/PB-3, PA/PB-4 were tested to be 213.91 kJ/kg, 14.86 kJ/kg, 42.82 kJ/kg, 57.54 kJ/kg and 83.81 kJ/kg, respectively. Additionally, for the PA/PB composites, the actual enthalpy was lower than the theoretical enthalpy because of the hydrogen bond between the PA chains and the porous PB, which could result in drag effect on the crystallization of PA molecules^24^.

**Table 2:** DSC data of the PA, PA/PB-1, PA/PB-2, PA/PB-3 and PA/PB-4.

Sample name	Melting		Freezing	
	Melting temperature T_m_ (°C)	Melting latent heat (kJ/kg)	Freezing temperature T_f_ (°C)	Freezing latent heat (kJ/kg)
Pure PA	62.07	219.63	61.79	213.91
PA/PB-4	59.25	84.74	59.13	83.81
PA/PB-3	58.77	58.56	58.24	57.54
PA/PB-2	58.11	43.62	57.61	42.82
PA/PB-1	57.49	14.88	57.33	14.86

Figure 11 shows the enthalpy curves of the PA/PB-4 before and after 50 cycles. The results showed that the fusing point and crystallization point of the PA/PB-4 after 50 cycles had a slight change compared with that of the PA/PB-4 before the thermal cycles. Moreover, the fusing enthalpy and crystallization enthalpy changed very little before and after 50 cycles. These changes had no significant impact on the thermal properties of the PA/PB-4. Therefore, the form-stable phase change material of PA/PB-4 prepared in this study had excellent thermal reliability.

Table 3 shows the thermal properties of form-stable phase change materials prepared with different supporters, and the results indicated that although the latent heat of PA/PB-4 prepared in this study was not the highest, it was competitive, and moreover, the supporter of PB was produced by forest residue of pinecone, which was cheap and environment friendly. Therefore, the PA/PB-4 had great potential for thermal energy storage applications.

**Table 3:** Thermal properties of different composite PCMs in literature.

Sample name	Melting temperature Tm (°C)	Latent heat (kJ/kg)	Concentration (wt%)	References
Myristic acid /high density polyethylene	53.8	109.9	60	Tang, *et al*.^46^
Diatomite/Palm Wax	55.95	77.14	—	Fort, *et al*.^47^
PEG2000/PAN	54	70	50	Sarier, *et al*.^48^
Tween-Span/nanoSiO_2_	60.33	53.77	20	Zhang, *et al*.^41^
PA/PB	59.25	84.74	60	Present study

### Thermal conductivity of PA and PA/PB-4

The thermal conductivity of PCMs, which could affect its thermal transfer efficiently, had an important influence on its applications^41^. The thermal conductivities of PA, PB and PA/PB-4 in this study were measured to be 0.2731, 0.3417 and 0.3926 W/(m∙K), respectively. The conductivity of PB was not very high, and however, it could improve the PA’s conductivity effectively for the reason that when PA was filled into the pores of PB, it could replace the air in the pores, and then the conductivity of PA/PB would be improved and higher than that of both PA and PB^42^. Additionally, the thermal conductivities of different form-stable phase change materials are listed in the Table 4. It could be seen that the conductivity of PA/PB-4 prepared in this study was not the highest, and however, it was favorite. Furthermore, the matrix of PB had the merits of cheap, extensive sources, environment friendly and easy to prepare. Hence, the biochar of PB produced from forest residue of pinecone was an ideal supporting material for the preparation of form-stable phase change materials.

**Table 4:** The thermal conductivity of different PCMs.

Sample name	Thermal conductivity W/(m∙K)	Concentration (wt%)	References
N-eicosane/CNT^a^	0.32	60	Karaipekli, *et al*.^49^
CA-MA^b^/PMMA^c^	0.269	—	Meng, *et al*.^50^
CA-SA^d^/WCB^e^	0.38	72.7	Liu, *et al*.^51^
PA/PANI^f^	0.32	75	Zeng, *et al*.^52^
PA/CNTs	0.292	99	Xiao, *et al*.^53^
PA/OCNTs^g^	0.298	99	Xiao, *et al*.^53^
PA/EG^h^	0.60	80	Sari, *et al*.^54^
PA/PB-4	0.3926	60	Present study

^a^carbon nanotube; ^b^capric-myristic acid; ^c^poly-methyl methacrylate; ^d^capric-stearic acid; ^e^white carbon black; ^f^polyaniline; ^g^oxidized carbon nanotubes; ^h^expanded graphite.

## Conclusion

A promising form-stable phase change material of PA/PB was prepared by vacuum impregnation operation using pinecone biochar of PB as the matrix of palmitic acid. The morphology, microstructure and thermal properties of PA/PB were studied. The results indicated that the PA was adsorbed well in the PB with porous structures because of the capillary force and surface tension between the PA and the porous PB, and there was no chemical reaction between the PA and PB. The enthalpies of the fusing and freezing of PA/PB-4 were 84.74 kJ/kg and 83.81 kJ/kg, respectively, and the thermal conductivity of PA/PB-4 was increased by 43.76% compared with that of the pure PA. Furthermore, the biochar PB, which was produced by forest residue of pinecone, was cheap, environmental friendly and easy to prepare. Therefore, the PA/PB prepared in this study had great potential for thermal energy storage applications.
